# Stress-strain relationship in pulmonary cells under bidirectional stretch application

**DOI:** 10.1186/cc9614

**Published:** 2011-03-11

**Authors:** K Gamerdinger, S Schumann, F Wernet, E Faehnrich, M Schneider, J Guttmann

**Affiliations:** 1 University Medical Center, Freiburg, Germany

## Introduction

Analysing the effects of mechanostimulation on pulmonary cells improves the understanding of the stress-strain relationship in the lungs. While there are plenty of different methods to apply strain on cells and thereby to analyze intracellular and extracellular processes, it remains difficult to measure the resulting strain, in other words the forces produced by cells to counteract the applied strain. Recently we presented a bioreactor to cyclically deflect cells by co-deflecting them with a carrier membrane [1]. The air-tight highly pliant siloxane-carrier membranes [2] used in our bioreactor were modified with Sulfo-SANPAH and RGD peptide [3] to allow cell adherence. Here we present actual data demonstrating changes in mechanical properties of pulmonary cell monolayers as a response to strain levels of up to 20% surface increase.

## Methods

Different alveolar epithelial cell lines (A549 and RLE-6TN) were grown on RGD-coated, highly flexible polydimethyl siloxane membranes and were mechanically stimulated in a bioreactor [1,2]. After becoming 100% confluent, microscopic images of cell monolayers were taken before subjecting them to increasing sinusoidal mechanical strain of up to 20% surface increase. The resulting stress was measured as the force that the cells opposed to the applied strain. Immediately after the procedure, additional images of cells were taken.

## Results

Stretching pulmonary cells bidirectionally led to a loss of intercellular connections and/or loss of integrin-binding sites to the RGD-labeled carrier membranes as indicated by comparing microscopic images before and after application of strain to cell monolayers. This was accompanied by a loss of the cell's counterforce on strain.

## Conclusions

The investigation of cell forces with our strain applicator allows us to analyze mechanical properties of cell constructs at the same time as we can track visually changes in cellular morphology. Strain-related cell damages as found in this study could play a role in development of ventilator-induced lung injury.
